# Rising plasminogen activator inhibitor-1 and hypoadiponectinemia characterize the cardiometabolic biomarker profile of women with recent gestational diabetes

**DOI:** 10.1186/s12933-018-0776-y

**Published:** 2018-10-09

**Authors:** Sadia Mehmood, Chang Ye, Philip W. Connelly, Anthony J. Hanley, Bernard Zinman, Ravi Retnakaran

**Affiliations:** 1 Leadership Sinai Centre for Diabetes, Mount Sinai Hospital, 60 Murray Street, Suite-L5-039, Mailbox-21, Toronto, ON M5T3L9 Canada; 2 Division of Endocrinology, University of Toronto, Toronto, ON Canada; 3 Keenan Research Centre for Biomedical Science of St. Michael’s Hospital, Toronto, Canada; 4 Department of Nutritional Sciences, University of Toronto, Toronto, ON Canada; 5 Lunenfeld-Tanenbaum Research Institute, Mount Sinai Hospital, Toronto, Canada

**Keywords:** Gestational diabetes, Adiponectin, Plasminogen activator inhibitor-1, Chemerin, CRP, RBP-4

## Abstract

**Background:**

Gestational diabetes (GDM) and milder gestational impaired glucose tolerance (GIGT) identify women at risk of developing type 2 diabetes and cardiovascular disease later in life. Accordingly, the postpartum years after gestational dysglycemia can provide insight into early events in the natural history of these disorders. We thus sought to prospectively evaluate the relationship between gestational glucose tolerance and emerging cardiometabolic biomarkers [adiponectin, chemerin, retinol-binding protein-4 (RBP-4), C-reactive protein (CRP), plasminogen activator inhibitor-1 (PAI-1)] at both 1- and 3-years postpartum in a cohort reflecting the full spectrum of gestational dysglycemia (from normal to GIGT to GDM).

**Methods:**

Three-hundred-and-thirty-nine women completed a glucose challenge test (GCT) and oral glucose tolerance test (OGTT) in pregnancy, which identified 4 gestational glucose tolerance groups: GDM (n = 105); GIGT (n = 59); abnormal GCT with normal OGTT (n = 99); and normal GCT with normal OGTT (n = 76). At 1- and 3-years postpartum, the women underwent repeat OGTT with measurement of biomarkers (adiponectin/chemerin/RBP-4/CRP/PAI-1).

**Results:**

Serum adiponectin was lower in women with GDM and GIGT at both 1-year and 3-years (both P ≤ 0.002), whereas chemerin, RBP-4, CRP and PAI-1 showed no differences across the 4 groups. Importantly, the change in PAI-1 between 1- and 3-years progressively increased from the normal GCT group to the abnormal GCT group to GIGT to GDM (P = 0.03). Indeed, both GDM (t = 2.98, P = 0.003) and GIGT (t = 2.14, P = 0.03) independently predicted an increase in PAI-1 from 1- to 3-years postpartum.

**Conclusions:**

Hypoadiponectinemia and rising PAI-1 over time are early features of the cardiometabolic biomarker profile of women with recent gestational dysglycemia.

**Electronic supplementary material:**

The online version of this article (10.1186/s12933-018-0776-y) contains supplementary material, which is available to authorized users.

## Background

Pregnancy poses a stress test for many aspects of maternal physiology and can thereby provide insight into a woman’s future risk of disease later in life. This concept can be clearly illustrated with the example of gestational diabetes mellitus (GDM), the diagnosis of which identifies a population of women who are at high risk of ultimately progressing to type 2 diabetes (T2DM) in the years after the pregnancy [1, 2]. Moreover, this relationship extends across the full spectrum of gestational dysglycemia. Indeed, each degree of antepartum glucose intolerance [ranging from GDM to milder gestational impaired glucose tolerance (GIGT) to lesser dysglycemia to normoglycemia] identifies a proportionate level of future risk of T2DM (one that is highest in GDM, followed by GIGT etc.) [2, 3]. In recent years, it has become apparent that this spectrum of glucose intolerance in pregnancy also identifies an analogous gradient of future risk of cardiovascular disease (CVD) [4]. Notably, both GDM and milder gestational dysglycemia identify proportionate risks of CVD that can manifest clinically within 12 years after the pregnancy [5–7]. It thus emerges that the physiologic changes that occur in the years following a pregnancy complicated by gestational dysglycemia can provide insight into the early natural history of both T2DM and CVD in women [2, 4].

In the past decade, there has been intense interest in novel cardiometabolic biomarkers that relate to future risk of metabolic and vascular disease. These circulating markers include adipokines, such as adiponectin, chemerin and retinol-binding protein-4 (RBP-4), and inflammatory proteins, such as C-reactive protein (CRP) and plasminogen activator inhibitor-1 (PAI-1). Given their associations with future cardiometabolic risk, the status of these emerging biomarkers in women with a history of GDM is of interest. However, previous studies of adipokines and inflammatory proteins in this patient population have yielded conflicting results [8–18]. Notably, these studies have been variously limited by modest sample sizes, cross-sectional designs, variable degrees of covariate adjustment, and variability in comparators (without accounting for the potential heterogeneity of those classified as non-GDM, as reflected in the metabolic implications of GIGT). Furthermore, previous studies have not addressed the possibility that women with recent gestational dysglycemia may exhibit differential changes over time in these biomarkers, since they comprise a patient population that is very early in the natural history of cardiometabolic disease in whom this risk potential may be evolving over time. Thus, hypothesizing that these limitations have contributed to this inconclusive literature, we sought to prospectively evaluate the relationship between gestational glucose tolerance status and emerging cardiometabolic biomarkers (adiponectin, chemerin, RBP-4, CRP, PAI-1) at both 1- and 3-years postpartum in a well-characterized cohort of women reflecting the full spectrum of gestational dysglycemia and hence a broad range of future risk of T2DM and CVD.

## Methods

The study population consisted of women participating in a prospective observational cohort study in which we are investigating the relationship between glucose tolerance in pregnancy and metabolic function in the years following delivery. The study protocol has been previously described in detail [3, 19]. In brief, women are first recruited at the time of antepartum screening for GDM in late 2nd/early 3rd trimester and undergo metabolic characterization at recruitment in pregnancy and again at 3-months and 1-year postpartum. At the latter visit, they are recruited into a long-term observational cohort study in which participants undergo serial metabolic characterization bi-annually thereafter. The current analysis evaluates changes in emerging cardiometabolic biomarkers in the first 339 women who have completed their 3-year postpartum visit. The study protocol was approved by the Mount Sinai Hospital Research Ethics Board, and all participants provided written informed consent.

### Recruitment and determination of glucose tolerance status in pregnancy

At our institution, all pregnant women are screened for GDM at 24–28 weeks’ gestation by 50 g glucose challenge test (GCT), followed by a diagnostic oral glucose tolerance test (OGTT) if the GCT result is abnormal (defined as plasma glucose ≥ 7.8 mmol/L at 1-h after ingestion of 50 g glucose). For this study, healthy pregnant women were recruited either prior to or just after their GCT and all participants undergo a 3-h 100 g OGTT for determination of gestational glucose tolerance status, regardless of their GCT result. As previously described [3], the recruitment of women after an abnormal GCT serves to enrich the study population for those with gestational dysglycemia (e.g. GDM, GIGT). The GCT and OGTT enable stratification of participants into the following gestational glucose tolerance groups:

(i) GDM, defined by National Diabetes Data Group (NDDG) criteria [20] which require at least 2 of the following on the OGTT: fasting blood glucose ≥ 5.8 mmol/L, 1-h glucose ≥ 10.6 mmol/L, 2-h glucose ≥ 9.2 mmol/L, or 3-h glucose ≥ 8.1 mmol/L;
(ii) GIGT, defined by meeting only one of the above NDDG criteria;
(iii) Abnormal GCT with normal glucose tolerance (NGT), defined by an abnormal GCT followed by NGT on the OGTT (i.e. meeting none of the NDDG criteria);
(iv) Normal GCT NGT, defined by a normal GCT followed by NGT on the OGTT.

These 4 groups identify a gradient of future diabetic risk (highest in GDM, followed in turn by GIGT and abnormal GCT NGT) and predict distinct trajectories thereof in the first 3-years postpartum [3].

### Assessments at 1- and 3-years postpartum

Participants returned to the clinical investigation unit at both 1- and 3-years postpartum for cardiometabolic characterization. Each study visit was performed in the morning after overnight fast and included a 2-h 75-g OGTT, on which current glucose tolerance status was defined according to current Diabetes Canada clinical practice guidelines [21]. Pre-diabetes refers to impaired glucose tolerance (IGT), impaired fasting glucose (IFT), or combined IFG and IGT.

Adipokines were measured from fasting serum samples on each OGTT with the following ELISA assays: total adiponectin (Millipore, St. Charles, MO); chemerin (Millipore, St. Charles, MO); and RBP-4 (Alpco, Salem, NH). High-sensitivity CRP was measured by endpoint nephelometry using the Dade-Behring BN ProSpec and N high-sensitivity CRP reagent (Dade-Behring, Mississauga, Canada). PAI-1 was measured by ELISA from Novex Invitrogen (Life Technologies, Burlington, Canada), with intra-assay coefficient of variation of 3.7–5.0% and inter-assay coefficient of variation of 6.1–9.1%.

### Statistical analyses

**Fig. 1:**
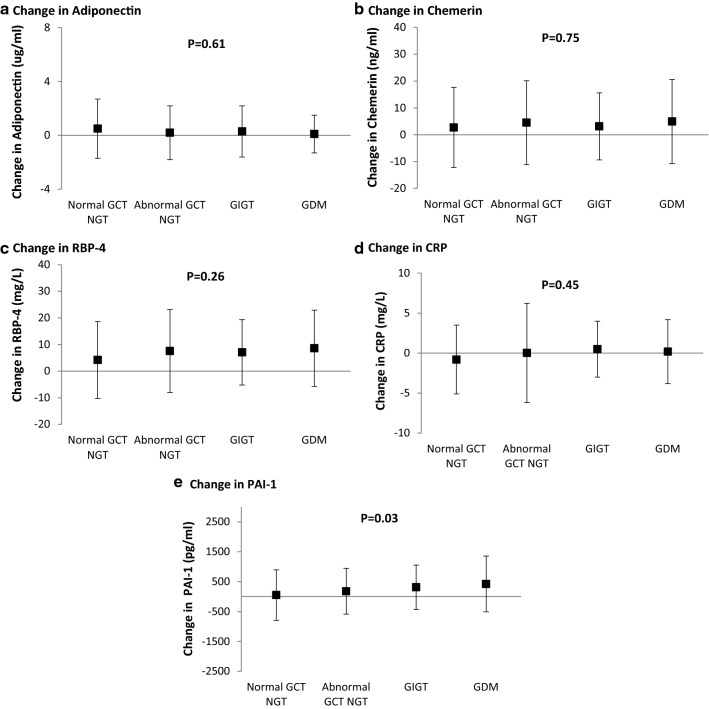
Comparison of gestational glucose tolerance groups with respect to the changes between 1- and 3-years postpartum in **a** adiponectin, **b** chemerin, **c** RBP-4, **d** CRP, and **e** PAI-1. P-values refer to overall comparison across the groups

All analyses were conducted using SAS 9.4 (SAS Institute, Cary, NC). All tests were two-sided and performed at a significance level of P < 0.05. Continuous variables were tested for normality of distribution. Variables with normal distributions are presented as mean ± standard deviation, and those with skewed distributions are presented as median and interquartile range (25th–75th percentile). Characteristics of the gestational glucose tolerance groups were compared at pregnancy, 1-year and 3-years postpartum by either one-way analysis of variance (ANOVA) for continuous variables (if normally-distributed) or Kruskal–Wallis test for continuous variables (if skewed), or either χ^2^ or Fisher exact test for categorical variables (Tables 1 and 2). The changes in cardiometabolic biomarkers from 1- to 3-years were normally-distributed and compared between the four gestational glucose tolerance groups by ANOVA (Fig. 1).

**Table 1:** Characteristics of study population in pregnancy and at 1-year postpartum, stratified into the following 4 groups based on gestational glucose tolerance status: normal GCT NGT, abnormal GCT NGT, GIGT, and GDM

	Normal GCT NGT	Abnormal GCT NGT	GIGT	GDM	P
	(n = 76)	(n = 99)	(n = 59)	(n = 105)	
In pregnancy					
Age (years)	35 ± 4	35 ± 4	34 ± 4	35 ± 4	0.71
Ethnicity					0.39
White (%)	75.0	74.7	67.8	65.8	
Asian (%)	6.6	9.1	11.9	17.1	
Other (%)	18.4	16.2	20.3	17.1	
Family history of T2DM (%)	54.0	59.6	64.4	64.8	0.46
Fasting glucose on OGTT (mmol/L)	4.3 ± 0.4	4.4 ± 0.3	4.6 ± 0.5	4.9 ± 0.7	*< 0.0001*
1-h glucose on OGTT (mmol/L)	7.7 ± 1.2	8.4 ± 1.3	10.2 ± 1.3	11.3 ± 1.6	*< 0.0001*
2-h glucose on OGTT (mmol/L)	6.7 ± 1.1	7.4 ± 1.0	8.4 ± 1.1	10.4 ± 1.5	*< 0.0001*
3-h glucose on OGTT (mmol/L)	5.9 ± 1.2	5.8 ± 1.3	7.2 ± 1.3	8.1 ± 1.7	*< 0.0001*
At 1-year postpartum					
Months breastfeeding (months)	11 (6–12)	10 (6–12)	9 (4–12)	10 (3–12)	0.36
BMI (kg/m^2^)	24.4 (21.5–28.4)	23.6 (21.8–27.8)	25.4 (23.1–30.1)	25.4 (22.5–29.4)	0.12
Waist circumference (cm)	87 ± 12	85 ± 11	89 ± 13	89 ± 14	0.05
Fasting glucose on OGTT (mmol/L)	4.6 ± 0.3	4.6 ± 0.4	4.9 ± 0.6	4.9 ± 0.5	*< 0.0001*
2-h glucose on OGTT (mmol/L)	5.3 ± 1.2	6.1 ± 1.5	6.4 ± 1.8	7.0 ± 1.9	*< 0.0001*
Current glucose tolerance status					*< 0.0001*
Normal (%)	97.2	87.4	77.8	69.7	
Pre-diabetes/diabetes (%)	2.8	12.6	22.2	30.3	
Adiponectin (μg/mL)	10.0 ± 4.0	9.8 ± 3.9	8.2 ± 3.1	8.5 ± 2.9	*0.002*
Chemerin (ng/mL)	55.8 ± 12.7	55.3 ± 14.8	58.6 ± 13.9	54.8 ± 16.0	0.48
RBP-4 (mg/L)	35.8 ± 14.8	37.5 ± 13.3	34.9 ± 10.3	35.3 ± 9.9	0.56
CRP (mg/L)	1.2 (0.6–3.7)	1.0 (0.5–2.9)	1.3 (0.6–2.7)	1.1 (0.7–3.1)	0.92
PAI-1 (pg/mL)	7354 (6964–7683)	7308 (6914–7667)	7114 (6558–7612)	7175 (6667–7540)	0.09

P-values refer to overall comparison across the 4 groups by either one-way analysis of variance or Kruskal–Wallis test for continuous variables, or either χ^2^ or Fisher exact test for categorical variables. Italic indicates P < 0.05

**Table 2:** Comparison of gestational glucose tolerance groups at 3-years postpartum

At 3-years postpartum	Normal GCT NGT	Abnormal GCT NGT	GIGT	GDM	P
BMI (kg/m^2^)	25.5 ± 4.5	25.3 ± 4.9	26.6 ± 4.8	26.8 ± 6.1	0.15
Waist circumference (cm)	88 ± 12	86 ± 12	89 ± 12	90 ± 13	0.15
A1c (%)	5.4 ± 0.3	5.5 ± 0.3	5.6 ± 0.3	5.6 ± 0.4	*0.0001*
Fasting glucose on OGTT (mmol/L)	4.6 ± 0.4	4.6 ± 0.5	4.8 ± 0.5	4.9 ± 0.6	*< 0.0001*
2-h glucose on OGTT (mmol/L)	5.5 ± 1.2	6.1 ± 1.7	6.6 ± 2.1	7.4 ± 2.2	*< 0.0001*
Current glucose tolerance status					*< 0.0001*
Normal (%)	93.4	85.9	79.7	63.8	
Pre-diabetes/diabetes (%)	6.6	14.1	20.3	36.2	
Adiponectin (μg/mL)	10.4 ± 4.6	10.1 ± 4.0	8.3 ± 3.6	8.6 ± 3.1	*0.0003*
Chemerin (ng/mL)	58.4 ± 14.0	59.7 ± 14.0	61.6 ± 13.7	59.9 ± 13.7	0.62
RBP-4 (mg/L)	39.7 ± 15.1	45.5 ± 18.4	42.2 ± 13.2	43.4 ± 18.0	0.16
CRP (mg/L)	1.1 (0.4–2.3)	0.9 (0.5–1.9)	1.0 (0.4–2.9)	1.5 (0.6–3.0)	0.09
PAI-1 (pg/mL)	7445 (6989–7724)	7366 (7109–7601)	7473 (7140–7654)	7446 (7193–7649)	0.43

P-values refer to overall comparison across the 4 groups by either one-way analysis of variance or Kruskal–Wallis test for continuous variables, or either χ2 or Fisher exact test for categorical variables. Italic indicates P < 0.05

Multiple linear regression analyses (Table 3) were performed to determine whether gestational glucose tolerance status was a significant independent predictor of the changes from 1- to 3-years postpartum for each of the following biomarkers: (Model I) adiponectin, (Model II) chemerin, (Model III) RBP-4, (Model IV) CRP, and (Model V) PAI-1. Each regression model included the following covariates: (i) diabetes risk factors (age, ethnicity, family history of diabetes, BMI at 1-year, duration of breastfeeding), (ii) glucose intolerance at 1-year, and (iii) gestational glucose tolerance status.

**Table 3:** Significant independent predictors of the respective changes from 1-year to 3-years postpartum in (I) adiponectin, (II) chemerin, (III) RBP-4, (IV) CRP, and (V) PAI-1

Model	Outcome	Significant predictors	Beta	t	P
I	Change in adiponectin	None	n/a	n/a	n/a
II	Change in chemerin	Asian ethnicity	− 10.6690	− 4.06	< 0.0001
		Glucose intolerance at 1-year	6.587417	2.85	0.005
III	Change in RBP-4	Age	0.426114	2.18	0.03
		Asian ethnicity	− 6.472599	− 2.47	0.01
IV	Change in CRP	Family history of diabetes	− 1.712047	− 3.13	0.002
		Non-white non-Asian ethnicity	1.813425	2.46	0.01
V	Change in PAI-1	GDM	416.1444	2.98	0.003
		GIGT	338.1283	2.14	0.03

Each model included the following covariates: age, ethnicity, family history of diabetes, BMI at 1-year, duration of breastfeeding, glucose intolerance at 1-year, and gestational glucose tolerance status

## Results

Table 1 shows the characteristics of the study population, stratified into the following 4 groups based on gestational glucose tolerance status: normal GCT NGT (n = 76), abnormal GCT NGT (n = 99), GIGT (n = 59), and GDM (n = 105). At recruitment, these groups did not differ in age, ethnicity, or family history of diabetes. As expected, at recruitment, there was a progressive rise in fasting glucose and each of 1-, 2-, and 3-h glucose from normal GCT NGT to abnormal GCT NGT to GIGT to GDM (all P < 0.0001).

At 1-year postpartum, the differences in fasting glucose and 2-h glucose persisted (both P < 0.0001), along with a stepwise increase across the 4 groups in the prevalence of dysglycemia (pre-diabetes or diabetes) from 2.8% to 12.8% to 22.2% to 30.3% (P < 0.0001). Amongst the emerging cardiometabolic biomarkers, only adiponectin differed across the groups at 1-year postpartum (P = 0.002), with lower concentrations in women with previous GDM and GIGT. Chemerin, RBP-4, CRP and PAI-1 did not differ across the 4 groups.

Similar findings were noted at 3-years postpartum (Table 2). Again, glycemia (A1c, fasting glucose, 2-h glucose) and the prevalence of pre-diabetes/diabetes progressively increased from normal GCT NGT to abnormal GCT NGT to GIGT to GDM (all P < 0.0001). As before, amongst the cardiometabolic biomarkers, only adiponectin differed across the groups (P = 0.0003), with lower levels observed in women with previous GDM and GIGT. There were no significant differences between the groups in chemerin, RBP-4, CRP and PAI-1. Moreover, these findings were unchanged after adjustment for diabetes risk factors (age, ethnicity, family history of diabetes, current BMI, duration of breastfeeding) and current glucose tolerance status. Specifically, mean adjusted adiponectin continued to differ across the groups (P = 0.01), with lower levels in women with previous GDM and GIGT, while mean adjusted chemerin, RBP-4, CRP and PAI-1 showed no significant differences (data not shown).

### Changes in cardiometabolic biomarkers between 1- and 3-years postpartum

Since differences in biomarkers between the gestational glucose tolerance groups may emerge over time, we next sought to compare these groups with respect to their changes in adiponectin, chemerin, RBP-4, CRP and PAI-1, respectively, from 1- to 3-years postpartum (Fig. 1). These analyses revealed that the change in PAI-1 between 1- and 3-years progressively increased from the normal GCT NGT group to the abnormal GCT NGT to GIGT to GDM (P = 0.03), whereas the concurrent changes in adiponectin, chemerin, RBP-4 and CRP did not differ across the groups (Fig. 1). Moreover, after adjustment for diabetes risk factors (age, ethnicity, family history of diabetes, BMI at 1-year, duration of breastfeeding) and glucose intolerance at 1-year, the mean adjusted change in PAI-1 between 1- and 3-years remained significantly different between the 4 groups (P = 0.019) (data not shown).

Finally, we performed multiple linear regression analyses of the changes from 1- to 3-years postpartum in adiponectin, chemerin, RBP-4, CRP, and PAI-1, respectively, in order to determine whether gestational glucose tolerance status was a significant independent predictor thereof. As shown in Table 3, gestational glucose tolerance status emerged as a significant predictor of the change in PAI-1, but not the change in any of the other biomarkers. Specifically, both GDM (t = 2.98, P = 0.003) and GIGT (t = 2.14, P = 0.03) independently predicted an increase in PAI-1 from 1- to 3-years postpartum.

We also performed sensitivity analyses in which the model of the change in PAI-1 from 1- to 3-years was further adjusted for the concurrent changes in fasting insulin and triglycerides, respectively (Additional file 1: Table S1). Upon further adjustment for the change in fasting insulin, GDM remained a significant predictor of the change in PAI-1 (t = 2.94, P = 0.0036), as did GIGT (t = 2.09, P = 0.037) (Additional file 1: Table S1A). Similarly, upon adjustment for the change in triglycerides, GDM was again a significant predictor (t = 2.94, P = 0.0036), while GIGT was associated at borderline significance (t = 1.88, P = 0.06) (Additional file 1: Table S1B).

## Discussion

In this study, we demonstrate that women with recent GDM and GIGT have lower serum adiponectin concentrations than their peers at both 1- and 3-years postpartum, with no concurrent dysregulation of chemerin, RBP-4, CRP and PAI-1. Importantly, the change in circulating levels of PAI-1 over this 2-year interval progressively increased across the gestational glucose tolerance groups (from normal GCT NGT to abnormal GCT NGT to GIGT to GDM), thereby tracking with the gradients of future cardiometabolic risk that these groups identify. Indeed, both GDM and GIGT independently predict an increase in PAI-1 from 1- to 3-years postpartum. It thus emerges that hypoadiponectinemia and rising PAI-1 over time are early features of the cardiometabolic biomarker profile of women with recent gestational dysglycemia.

Previous studies have noted adipokine dysregulation and evidence of subclinical inflammation in women with a history of GDM [8–13], but have not been consistent, with some reporting conflicting findings in this regard [14–18]. Limitations of these studies have included modest samples sizes, cross-sectional evaluation at a single point in time, variability in comparators and variable degrees of covariate adjustment (including, most notably, the absence of adjustment for concurrent glucose intolerance). The current study was thus designed to address these limitations with prospective ascertainment of glucose tolerance status in pregnancy to establish a well-characterized cohort of 339 women across the full spectrum of gestational glycemia (from normal to GDM), who then underwent serial assessment of both adipokines/inflammatory proteins and glucose tolerance on two occasions in the first 3-years after delivery.

### Adiponectin and GDM

With this design, we demonstrate that, despite clear differences in glycemia between the previous gestational glucose tolerance groups, differences in adipokines and inflammatory proteins were limited to adiponectin only. This finding of low adiponectin is consistent with emerging lines of evidence suggesting that hypoadiponectinemia may be a chronic feature of women who develop GDM. First, women who develop GDM have lower adiponectin than their peers at diagnosis in pregnancy [22]. Second, hypoadiponectinemia in early pregnancy, or even prior to gestation, can predict the subsequent development of GDM in 2nd/3rd trimester [23–25]. Third, it has recently been demonstrated that genetic knock-out of adiponectin results in impaired beta-cell adaptation to pregnancy in mice, thereby yielding a murine model of gestational dysglycemia and potentially linking adiponectin to the pathophysiologic basis of GDM (insufficient beta-cell compensation for the insulin resistance of pregnancy) [26, 27]. Taken together with the current demonstration that women with recent GDM have low adiponectin at both 1- and 3-years after delivery, these data collectively suggest that hypoadiponectinemia is a chronic feature of this patient population before, during, and after pregnancy. Moreover, a higher leptin/adiponectin ratio in women with GDM has been associated with an unfavourable cardiovascular risk factor profile on postpartum follow-up [28] and hypoadiponectinemia in pregnancy has been suggested as a possible factor contributing to preeclampsia, which is also associated with future risk of CVD [29].

### PAI-1 and GDM

In contrast to adiponectin, serum chemerin, RBP-4, CRP and PAI-1 did not differ between the gestational glucose tolerance groups at either 1- or 3-years postpartum. As the current setting (young women within 3 years of delivery) is presumably very early in the natural history of cardiometabolic disease, we reasoned that other differences in these biomarkers between women with previous GDM and theirs peers potentially may emerge over time. In this context, our serial measurements at 1- and 3-years postpartum can offer insight. Indeed, the change in PAI-1 over this 2-year interval progressively increased from the normal GCT NGT group to abnormal GCT NGT to GIGT to GDM, thereby mirroring the gradient of future cardiometabolic risk that gestational glucose tolerance identifies [3]. Moreover, both GDM and GIGT emerged as independent predictors of rising PAI-1 over this time. Accordingly, these data raise the possibility that women with previous GDM and GIGT may ultimately exhibit higher PAI-1 than their peers with the further passage of time. The relationship between gestational dysglycemia and changes over time in serum PAI-1 in the years thereafter thus requires further study. The importance of future study in this regard is underscored by the increased lifetime risk of cardiometabolic disease that GDM and GIGT predict in affected women [4–7].

Though best known as an inhibitor of the fibrinolytic system, PAI is now recognized as having physiologic effects beyond hemostasis. Notably, in recent years, PAI-1 has emerged as a potential marker and mediator of cellular senescence associated with aging and aging-related pathologies [30]. Indeed, the recognition that increased circulating PAI-1 contributes to the multi-morbidity of aging may provide an underlying basis for its associations with cardiometabolic disease [30]. Specifically, higher PAI-1 has been associated with risks of both incident T2DM and CVD, though there has been debate as to whether it plays a causal role in either case [31, 32]. Of note, a recent pooled analysis of 8 prospective studies revealed that, compared to those in the lowest tertile, individuals in the highest tertile of PAI-1 at baseline had a 67% higher risk of T2DM over median follow-up of 5.7 years [31]. Furthermore, in the Insulin Resistance and Atherosclerosis Study (IRAS), both baseline PAI-1 and its change over time predicted incident T2DM [33, 34], with higher PAI-1 predicting deterioration of insulin clearance over 5 years (a relationship not seen with other biomarkers including CRP, tumor necrosis factor-α, leptin and fibrinogen) [35]. Similarly, in the early postpartum years, we have shown that rising PAI-1 is independently associated with lower insulin sensitivity [19]. In addition, Song and colleagues recently reported Mendelian randomization analyses suggesting a causal effect of higher circulating PAI-1 concentration on the risk of coronary heart disease that may be partly mediated by dysglycemia [32]. In this context, the current observation of differential changes in PAI-1 in young women with varying degrees of recent gestational dysglycemia raises the possibility that PAI-1 may be a very early biomarker for tracking progression towards the clinical manifestation of cardiometabolic risk in this patient population (i.e. T2DM and CVD). Further longitudinal study of the respective relationships over time between PAI-1 and both glucose tolerance and vascular function in young women is thus warranted.

### Limitations

A limitation of this study is that the patient population of young women in the early postpartum precludes evaluation of the associations of these biomarkers with hard clinical outcomes such as major cardiovascular events. In addition, besides those observed herein, it is possible that other differences between the study groups in adipokines and inflammatory proteins may emerge with the further passage of time. Conversely, however, our findings of stable hypoadiponectinemia and rising PAI-1 over 2-years have provided novel temporal insight into early biomarker changes and thereby identified analytes of interest for further longitudinal surveillance in future studies.

## Conclusions

In summary, women with recent GDM and GIGT have lower serum adiponectin concentrations than their peers at both 1- and 3-years postpartum, with no concurrent dysregulation of chemerin, RBP-4, CRP and PAI-1. Moreover, both GDM and GIGT independently predict an increase in PAI-1 from 1- to 3-years postpartum. It thus emerges that rising PAI-1 and hypoadiponectinemia are early features of the cardiometabolic biomarker profile of women with recent gestational dysglycemia that may relate to their future risks of T2DM and CVD.

## Additional file

**Additional file 1.** Multiple linear regression of (dependent variable) change in PAI-1 from 1- to 3-years adjusted for age, ethnicity, family history of diabetes, BMI at 1-year, duration of breastfeeding, glucose intolerance at 1-year, previous gestational glucose tolerance status, and the concurrent change in fasting insulin (Panel A) or triglycerides (Panel B).

## Abbreviations

CRP: C-reactive protein
CVD: cardiovascular disease
GCT: glucose challenge test
GDM: gestational diabetes mellitus
GIGT: gestational impaired glucose tolerance
NGT: normal glucose tolerance
PAI-1: plasminogen activator inhibitor-1
RBP-4: retinol-binding protein-4
T2DM: type 2 diabetes
