# Supplementary material for: Rising plasminogen activator inhibitor-1 and hypoadiponectinemia characterize the cardiometabolic biomarker profile of women with recent gestational diabetes
Source: Cardiovasc Diabetol. 2018 Oct 9;17:133. doi: 10.1186/s12933-018-0776-y (PMC6176527; doi:10.1186/s12933-018-0776-y)
Supplement: Supplementary file 1 — Additional file 1. Multiple linear regression of (dependent variable) change in PAI-1 from 1- to 3-years adjusted for age, ethnicity, family history of diabetes, BMI at 1-year, duration of breastfeeding, glucose intolerance at 1-year, previous gestational glucose tolerance status, and the concurrent change in fasting insulin (Panel A) or triglycerides (Panel B). [file 12933_2018_776_MOESM1_ESM.pdf]

Table S1: Multiple linear regression of (dependent variable) change in PAI-1 from 1- to 3-years adjusted for age, ethnicity, family history of diabetes, BMI at 1-year, duration of breastfeeding, glucose intolerance at 1-year, previous gestational glucose tolerance status, and the concurrent change in fasting insulin **(Panel A)** or triglycerides **(Panel B)**

Panel A:

Variables in model	Beta	t	P
Age	17.97186	1.56	0.12
Asian ethnicity	-90.75315	-0.59	0.56
Non-white non-Asian ethnicity	22.46827	0.16	0.87
Family history of diabetes	-138.3005	-1.37	0.17
BMI at 1-year	-4.54252	-0.45	0.65
Duration of breastfeeding	6.633913	0.64	0.52
Glucose intolerance at 1-year	47.93762	0.35	0.72
GDM	412.2271	2.94	0.0036
GIGT	333.7635	2.09	0.037
Abnormal GCT NGT in pregnancy	162.9566	1.19	0.24
Change in fasting insulin	0.677118	0.47	0.64

Panel B:

Variables in model	Beta	t	P
Age	12.71499	1.11	0.27
Asian ethnicity	-53.35009	-0.35	0.73
Non-white non-Asian ethnicity	52.73081	0.39	0.70
Family history of diabetes	-126.3989	-1.27	0.21
BMI at 1-year	-1.58756	-0.16	0.88
Duration of breastfeeding	7.182945	0.70	0.49
Glucose intolerance at 1-year	59.3471	0.44	0.66
GDM	405.5285	2.94	0.0036
GIGT	298.3018	1.88	0.06
Abnormal GCT NGT in pregnancy	128.9917	0.95	0.34
Change in triglycerides	50.40535	0.44	0.66
